# Evaluation of Phantom Doping Materials in Quantitative Susceptibility Mapping

**DOI:** 10.1002/mrm.70427

**Published:** 2026-06-25

**Authors:** Padriac Hooper, Jin Jin, Kieran O'Brien, Monique Tourell, Simon Daniel Robinson, Markus Barth

**Affiliations:** ^1^ ARC Training Centre for Innovation in Biomedical Imaging Technology Brisbane Australia; ^2^ Centre for Advanced Imaging The University of Queensland Brisbane Australia; ^3^ Siemens Healthcare Pty Ltd Brisbane Australia; ^4^ School for Electrical Engineering and Computer Science The University of Queensland Brisbane Australia; ^5^ High Field MR Center, Department of Biomedical Imaging and Image‐Guided Therapy Medical University of Vienna Vienna Austria; ^6^ Christian Doppler Laboratory for MR Imaging Biomarkers, Department for Biomedical Imaging and Image‐Guided Therapy Medical University of Vienna Vienna Austria

## Abstract

**Purpose:**

To measure magnetic susceptibility (*χ*) with Quantitative Susceptibility Mapping (QSM) and evaluate its repeatability using four phantom doping materials relevant to QSM applications.

**Methods:**

A cylindrical phantom was constructed containing vials of agarose gel doped with two paramagnetic materials (ferritin, USPIO) and two diamagnetic materials (CaCl_2_, CaCO_3_) at five concentrations each. Single orientation QSM measurements (MEDI+0) were carried out on the phantom at 3 and 7 T. We measured molar susceptibility (*χ*_mol_) from QSM and evaluated the precision of QSM measurements using the standard deviation of the ROI measurement (SD_ROI_). We evaluated material lifespan by conducting a *t*‐test of *χ*_mol_ at various timepoints.

**Results:**

*χ*_mol_ (ppm L mmol^−1^) were measured as 1.67 ± 0.24 and 0.74 ± 0.09 (USPIO: 3 and 7 T, respectively), 10^−2^ × (8.13 ± 1.35; 8.13 ± 1.19) (Ferritin: 3; 7 T), 10^−4^ × (−2.68 ± 0.24; −2.71 ± 0.37) (CaCl_2_: 3; 7 T), and 10^−5^ × (−9.52 ± 1.44; −9.53 ± 1.18) (CaCO_3_: 3; 7 T). We observed no significant changes in molar susceptibility for ferritin and CaCO_3_ over the measured timeframes (24 and 15 months, respectively).

**Conclusion:**

We recommend using ferritin as a paramagnetic dopant. Further research is required to identify a diamagnetic dopant with a lower electrical conductivity and a lower ratio of *R*_2_*/*B*_0_ to *χ*.

## 1 Introduction

MRI employs electromagnetic fields to excite and detect nuclear spin resonance. These spins act as probes of the local microenvironment, and with the appropriate model‐based analysis, provide a means for quantifying physical properties related to tissue structure and composition. Quantitative parameters (e.g., *T*_1_, *T*_2_, diffusion coefficients) can be derived for every voxel in an MRI image and can serve as biomarkers of disease profiles [1, 2]. The magnetic susceptibility of tissue, *χ*, is sensitive to both tissue structure and composition and can be measured using the phase component of the MRI signal acquired from *T*_2_*‐weighted images in Quantitative Susceptibility Mapping (QSM). Clinically, QSM has produced a groundswell of interest, finding applications in mapping calcifications [3], venous oxygenation [4], and iron content [5, 6]. However, several challenges remain in QSM imaging. Firstly, local *χ* variations (e.g., air‐tissue interfaces) are a source of field distortions and produce regions of low SNR [7], which lead to artifacts in the susceptibility map [8]. Secondly, the dipole kernel contains zeros in k‐space at spatial frequencies corresponding to that of a double cone [9, 10], making dipole inversion an ill‐posed problem necessitating regularization. Also, because of the absence of spatial frequencies at the center of k‐space, QSM requires referencing to a known susceptibility value, which is difficult to define in vivo [11]. Additionally, the susceptibility of some tissues are not scalar but tensor, and susceptibility is affected by tissue microstructure, for example, the radial anisotropy of the myelin sheath [12].

Susceptibility imaging phantoms that contain uniform regions of known susceptibility are not affected by patient/biological factors [13, 14, 15, 16]. As a result, they provide reliable reference values, assess errors in MR acquisition and QSM reconstructions, and enable calibration across scanners and imaging sites [17, 18]. In vivo, the predominant cause of tissue susceptibility are iron‐ or calcium‐containing materials that produce paramagnetic (positive) or diamagnetic (negative) susceptibility contrast, motivating the use of iron‐based or calcium‐based materials, respectively, as *χ* sources in phantom inclusions. Most phantom inclusions are composed of gel mixtures in place of aqueous mixtures, since gels mimic the in vivo relaxation properties of soft tissues [19] and embed particles in a fixed position after solidification. Mimicking the signal relaxation and susceptibility of iron‐ and calcium‐based materials provides a realistic evaluation of QSM reconstructions. The protein ferritin, is biologically relevant as a dominant form of iron stored within deep gray matter [20]. To mimic ferritin for QSM applications, Cuna et al. synthesized an iron‐filled hydrogel phantom material with variable cluster size, and a comparable molar susceptibility to ferritin in vivo [18]. The same research group evaluated QSM susceptibility measurements with a SQUID magnetometer, showing the iron‐filled hydrogel phantom material had comparable measurements to those acquired with ultra‐high‐field scanners [21]. An iron‐filled hydrogel phantom material bears a resemblance to iron clustering systems, which are observed Alzheimer's beta‐amyloid plaques [5, 6]. Another paramagnetic material of interest are ultrasmall particles of iron oxide (USPIO), which are applied to QSM as a blood‐pool contrast agent [4, 22, 23] and in magnetic fluid hyperthermia [24]. USPIO have been used to validate *R*_2_* mapping at clinical field strengths [25], and USPIO *R*_2_* is invariant across clinical field strengths [4]. An important material observed in vivo are the insoluble polyhedral salt crystals, calcium carbonate (CaCO_3_), which are present in biochemical analyses within bone mineralization and calcifications [26, 27, 28]. Emmerich et al. evaluated CaCO_3_ particles using clinical and ultra‐high‐field scanners, indicating that CaCO_3_ *R*_2_*/*B*_0_ is invariant across field strengths [29]. Using this knowledge, Emmerich et al. used CaCO_3_ particles to study the separation of *χ* sources in QSM [30], with in vivo comparison to multiple sclerosis lesions [31]. An alternative diamagnetic *χ* source is calcium chloride (CaCl_2_), which has been used previously by Hopkins et al. to match the susceptibility of bone [32], and is of practical utility in a phantom due to its high diamagnetism, water solubility and inert chemical properties [33].

This study aimed to evaluate the repeatability of MR‐based *χ* measurements of two paramagnetic and two diamagnetic materials: USPIO, ferritin (paramagnetic), CaCl_2_ and, CaCO_3_ (diamagnetic). To do this, a cylindrical phantom was constructed containing vials of doped agarose gel. Susceptibility measurements were reconstructed from GRE data at 3 and 7 T, the precision of susceptibility measurements were assessed, then evaluated over a 9‐ to 24‐month period. Based on our results we draw conclusions on the suitability of doping materials in QSM phantom studies.

## 2 Methods

### 2.1 Phantom Design

Relevant design factors for producing the QSM phantom: it should (i) fit within most RF head coils, (ii) produce minimal *B*_0_ and *B*_1_ inhomogeneities, (iii) provide a means to trap air bubbles, and (iv) be stable over the long‐term (≥ 12 months).

**FIGURE 1:**
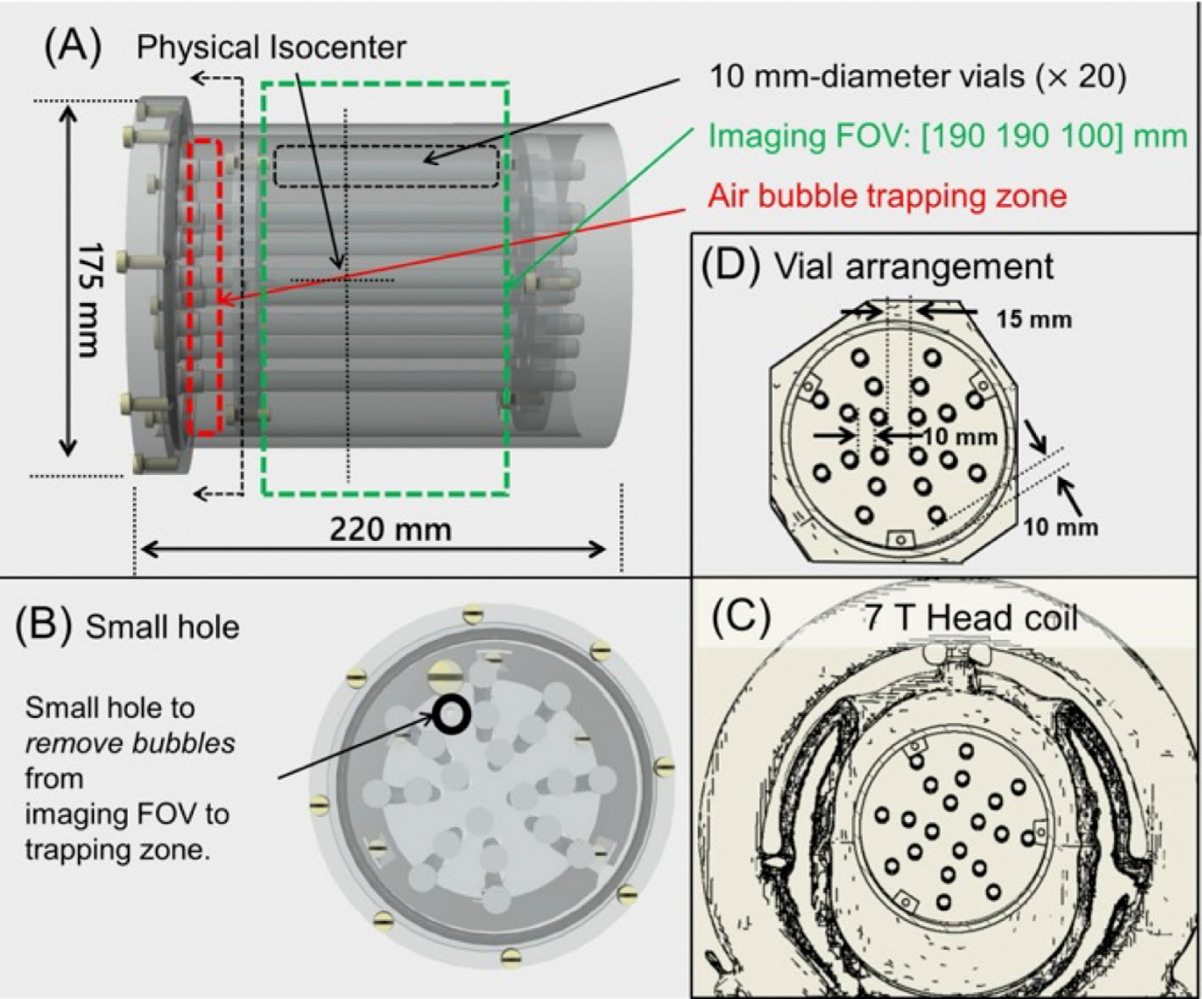
Side (A) and top (B) views of phantom. (C) CAD drawing of the phantom within an approximation of the 7 T Nova Medical 32 Rx coil. (D) CAD drawing indicating relative positioning of vials.

The phantom schematics are shown in Figure 1. To ensure fitting in what is probably the most widely used head coil at ultra‐high‐field currently, the 7 Tesla Nova Medical 1Tx/32Rx head coil (Nova Medical, Wilmington, MA), the outer diameter of the phantom was 150 mm. As most other head coils—including other field strengths—are larger, they would be able to accommodate a phantom of this size. The phantom contains 20 NMR vials (10 mm outer diameter, N‐51A, Kimble Glass) of doped agarose gel (details below) surrounded by a solution of ultrapure water (Milli‐Q IQ 7000, Merck & Co.) 44.3 weight percentage (wt.%) PVP‐40 (PVP‐40, Merck & Co.) and 1.7 wt.% NaCl (746398, Merck & Co.) to mimic the electric permittivity and conductivity of white matter at 7 T. To reduce *B*_1_ artifacts and errors during background field correction, vials were positioned at least 10 mm away from the phantom periphery. The phantom was designed to accommodate a bubble trapping compartment outside the Field of View (FOV), connected to the main compartment by a small hole (see Figure 1a). The tight‐fitting non‐magnetic closure and an NMR tube cap prevented microbes from entering the phantom and samples, respectively. The modification of electric permittivity in the phantom was important to prevent standing wave artifacts at 7 T that would lead to image inhomogeneities.

The phantom design features four quadrants, each containing five vials. The samples were prepared by doping 1.5 wt.% hot agarose gel (A4018, Merck & Co.) with one of the four dopants: USPIO (Molday Ion, BioPAL, 10 mg Fe/mL), ferritin (F4503, Merck & Co., 8.5 mg Fe/mL), CaCl_2_ (746495, Merck & Co.) and CaCO_3_ (C4830, Merck & Co.) at five equally spaced concentrations. Further detail on agarose gel preparation is provided in a PhD thesis [34]. Other relevant dopants, Gd‐DTPA [35], hydroxyapatite [36], and tungsten carbide [30] were not covered in this study. The concentrations were chosen to more than cover the magnetic susceptibility range expected for in vivo human brain scans, with USPIO ranging from 0.18 to 0.54 mmol/L, ferritin from 3.76 to 10.21 mmol/L, CaCl_2_ from 0.9 to 4.5 mol/L and, CaCO_3_ from 1.0 to 5.0 mol/L. Ultrapure water (Milli‐Q IQ 7000, Merck & Co.) was used as a solution for the PVP‐NaCl mixture to limit the likelihood of microorganisms or magnetic ions entering the samples. The phantom was given at least 48 h for the PVP‐NaCl mixture to settle before scanning [37].

### 2.2 MR Acquisition

The phantom was scanned at two different field strengths of 3 and 7 T (all Siemens Healthcare, Erlangen, Germany) with a 20‐channel and 32‐channel head coil respectively. Phase and magnitude images were acquired with a multi‐echo 3D GRE pulse sequence using the parameters shown in Table 1. We used bipolar acquisitions with shortest possible echo spacings as the results of phase unwrapping are generally more reliable when the shortest possible echo spacings are used [38, 39] because SNR is high and wraps fewer in number [40]. *B*_0_ shimming was performed using Siemens' GRE brain sequence and Prescan normalize correction [41] was used with the 20‐channel head coil at 3 T. Images were reconstructed using ASPIRE [38] for phase and root sum‐of‐squares magnitude. For SNR measurement, unfiltered magnitude images (parallel imaging, partial Fourier and, elliptical scanning turned ‘off’) were reconstructed. The DICOMs were then converted to NIFTI [42] format for QSM processing. The phantom was scanned twice at baseline (*t*_0_), again after 9 months (*t*_1_), then again twice at 24 months (*t*_2_). The test–retest scans were acquired within 15 min of one another. Some materials were included and removed from the phantom at different timepoints; USPIO and ferritin included at *t*_0_, *t*_1_ and *t*_2_; CaCl_2_ included at *t*_0_, *t*_1_; CaCO_3_ included at *t*_1_, *t*_2_.

**TABLE 1:** MR acquisition parameters used in this study.

	3 T	7 T
Model	Magnetom Prisma	Magnetom 7 T Plus
Gradient readout	Bipolar	
TE_1_:∆TE:TE_max_; TR (ms)	1.87:1.87:22.44; 26	3.15:3.15:28.35; 32
Coil	20‐ch Rx head coil	32‐ch Rx head coil
Voxel size	1.0 mm^3^ isotropic	0.7 mm^3^ isotropic
Acquisition matrix	192 × 192 × 112	272 × 272 × 160
Flip angle; Bandwidth; Averages	15°; 1000 Hz/pix; 1	15°; 340 Hz/pix; 1
Acceleration? type and factor	✗	GRAPPA 2
Partial Fourier? factor; Elliptical scanning?	✗;✗	6/8 PFA; ✓
Prescan normalize correction?	✓	✗

### 2.3 Image Pre‐Processing and Corrections

Noise was measured from unfiltered magnitude images as the standard deviation of the intensity in air regions outside the phantom. Regions of interest (ROIs) 20 × 20 × 160 mm^3^ were drawn manually at the image corners, ensuring ROIs were free of artifacts and matrix borders. The expected noise level was measured by dividing the standard deviation of air regions by 0.66 [43]. The signal was measured using cylindrical ROIs (7 mm diameter) drawn manually within each vial, excluding partial volume and/or signal loss at the vial edge, and excluding aliasing artifacts at the 15 distal slices on either end.

The criterion for echo time selection for QSM is given: all echo times with SNR less than or equal to 10:1 was excluded. No echo times were excluded for USPIO, ferritin, CaCl_2_; however, echo times later than 11 ms at 3 T and 3 ms at 7 T (see Table S1) were excluded for CaCO_3_. For *R*_2_* mapping with CaCO_3_ at 7 T, we excluded echo times later than 9 ms.

To correct for slight differences in the phantom orientation and positioning between longitudinal acquisitions, the real and imaginary images were manually co‐registered (ITK‐SNAP v4.0 [44]) using the first echo magnitude as a reference, interpolating using B‐splines. There were phase offsets associated with opposite polarity of odd and even echoes for a bipolar acquisition [45], which were corrected using MCPC‐3D‐S [38]. Susceptibility‐induced geometric distortions occur in the readout direction of bipolar GRE acquisitions due to the opposing direction of odd and even readout gradients [46]. Note that geometric distortions were not observed at 3 T due to the high readout pixel bandwidth (as indicated in Table 1). Field maps of odd and even echoes were created, combining the phase using nonlinear complex fitting [47], then unwrapped using SPURS [48]. The voxel displacement map was calculated by dividing the field map by the readout pixel bandwidth [49]. The warped field was generated by applying smoothing to the voxel displacement map [50]. The real and imaginary images were unwarped (SPM12) [51] with tri‐linear interpolation. Gibbs artifacts associated with sharp transitions in signal intensity were observed at the vial boundary. A Gibbs ringing correction was applied to the real and imaginary images using sub‐voxel shifts in all three spatial dimensions [52, 53].

### 2.4 *R* _2_ * Mapping, Analysis of Dopant Clumping

Maps of transverse relaxation (*R*_2_*) were generated by voxel‐wise nonlinear fitting of the multi‐echo magnitude image using the Levenberg‐Marquardt algorithm. Within the doped gel mixture, regions of reduced signal (due to increased *R*_2_*) can arise due to ‘clumps’ of concentrated dopant and can extend beyond the clump (‘blooming artifacts’). Regions of elevated *R*_2_* were selected as follows: (1) determining local outliers (median + 3·IQR), then (2) finding continuous (6‐connectivity) regions of outliers larger than 1 mm^3^. For each material group, we reported (i) the volume of increased *R*_2_* in mm^3^, and (ii) the percentage of increased *R*_2_* per ROI.

### 2.5 QSM

An ‘initial mask’ was generated by thresholding values within the first‐echo magnitude image greater than 2% of its maximum intensity. A morphological opening operation, which removes disconnected voxels from the mask, was applied using a structural spherical element of 5 mm radius.

Zero‐padding of the complex GRE data prior to non‐linear field map estimation is known to result in a more accurate field map [54]. Moreover, it is well established that zero‐padding reduces aliasing associated with the Fourier Transform, which affects both background field correction and dipole inversion stages of QSM processing. Therefore, the imaging matrices were zero‐padded (factor of 1.5) from the original FOV 192 × 192 × 112 mm^3^.

The phase was combined using nonlinear complex fitting [47], then unwrapped using SPURS [48]. Background fields were then corrected using V‐SHARP [55], SMV‐radii = 1:1:10 mm (rounding to the nearest integer towards infinity). The maximum SMV radius was set to 10 mm since the vials positioned close to the perimeter were 10 mm from the mask edge, which is a limitation of V‐SHARP [56]. Including the spatial distribution of all frequencies and *χ* sources improves background field correction [57], therefore, the mask imposed on the field map (and on *χ* during dipole inversion) was set as the entire matrix.

The ‘weighting map’ (used to weigh the data consistency term) was computed as described in SEPIA documentation (https://sepia‐documentation.readthedocs.io/en/latest/method/weightings.html) [58]. The field noise map was inverted, normalized using the median and upper IQR, re‐centered to 1, then global outliers (defined as median + 3·IQR) were replaced with a 3 × 3 × 3 voxel box filtered copy. The relative residual (Equation 3) of *S*_measured_ (Equation 1) and *S*_simulated_ (Equation 2) was computed as follows,

(1) $$S_{\text{measured}}=S\left(\mathrm{TE}\right)\cdot e^{-j\cdot \phi_{S\left(TE_{1}\right)}}$$

(2) $$S_{\text{simulated}}=S_{0}\cdot e^{-R_{2}^{*}\cdot \mathrm{TE}+j\cdot \omega \cdot \mathrm{TE}-j\cdot \omega \cdot TE_{1}}$$

(3) $$\text{relativeresidual}=\frac{\sum^{\mathrm{TE}{\left|S_{\text{simulated}}\left(\mathrm{TE}\right)-S_{\text{measured}}\left(\mathrm{TE}\right)\right|}^{2}}}{\sum^{\mathrm{TE}{\left|S_{\text{measured}}\left(\mathrm{TE}\right)\right|}^{2}}}$$

In these equations, *ω* was the angular frequency determined during field mapping, *S*_0_ was the extrapolated signal magnitude at TE = 0, *S*_measured_ was the measured data with the phase subtracted from the first echo, and *S*_simulated_ was the simulated mono‐exponential model signal with the phase subtracted from the first echo. The relative residual map was brought into a weighting component using a threshold of 0.3, which was then used to modulate the weighting map [58]. The modulation of the weighting map using Equations ((1), (2), (3)) was not applied for the 7 T CaCO_3_ data for which only single echo data was used.

MEDI+0 [59, 60] was performed for dipole inversion,

(4) $$\underset{\chi }{\text{argmin}}{\left\|W\cdot \left(e^{\mathrm{i\delta}-i\left(d⊗\chi \right)}\;\right)\right\|}_{2}^{2}+\lambda_{1}{\left\|E\nabla \chi \right\|}_{1}+\lambda_{2}{\left\|M_{R}\left(\chi -{\bar{\chi }}_{R}\right)\right\|}_{2}^{2}$$

In which *δ* and *d* denoted local fields and dipole kernel, respectively. The term $\lambda_{2}{\left\|M_{R}\left(\chi -{\bar{\chi }}_{R}\right)\right\|}_{2}^{2}$ penalizes susceptibility variation within the reference region, implicitly setting ${\bar{\chi }}_{R}$ to zero. The reference region was isolated using a ‘reference mask’ ($M_{R}$), which was obtained in three steps: (1) *R*_2_* thresholding at 5 s^−1^, (2) morphological closing (3 mm radius), then (3) morphological erosion (10 mm radius), as indicated in Figure S1. In the second term, ∇ denoted the gradient operator, and E denoted the edge weighting mask derived from the gradient magnitude image (∇m) by considering a given ratio of voxels (c_∇_ = [0,3]) to be edges [61]:

(5) $$\begin{array}{c} E=\left\{\begin{array}{c} 0\left|\nabla m\right|\geq c_{\nabla }\\ 1\left|\nabla m\right|<c_{\nabla } \end{array}\right. \end{array}$$

It was determined that c_∇_ = 0.5 gave a balance between morphological consistency and image fidelity (see Figure S3).

### 2.6 Automatic Segmentation

We performed automatic segmentation instead of manual segmentation to obtain clear and consistent cylindrical ROIs for statistical analysis. A mask of *R*_2_* values greater than 5 s^−1^ (to exclude the background fluid) was multiplied by the ‘initial mask’ (eroded by 10 mm to remove voxels on the phantom edge), obtaining a mask containing the doped‐agarose materials. To exclude erroneous voxels at the vial edge, the obtained mask was eroded by 4 mm (reducing ROI diameter from 10 to 6 mm), then morphologically opened by 1 mm (filling in small holes). We also excluded the 15 distal slices on either end which were prone to aliasing artifacts. Segmentation was performed on the obtained mask using the cluster function within FSL [62].

### 2.7 Statistical Analysis

The susceptibility maps were restored back to the original FOV (192 × 192 × 112 mm^3^). The molar concentration (*c*_mol_) was fitted against the mean ROI measurement (*R*_2_* or *χ*) with a least‐squares regression to determine the linear fit:

(6) $$\begin{array}{c} R_{2}^{*}={R_{2}^{*}}_{0}+c_{\mathrm{mol}}\cdot {R_{2}^{*}}_{\mathrm{mol}} \end{array}$$

(7) $$\begin{array}{c} \chi =\chi_{0}+c_{\mathrm{mol}}\cdot \chi_{\mathrm{mol}} \end{array}$$

We used robust regression with a bisquare weighting function, which reduces the weight of independent variables with a high least‐squares residual. *R*_2_*_0_; *χ*_0_ are estimates of zero dopant *R*_2_*; *χ*, respectively. *R*_2_*_mol_; *χ*_mol_ are equal to the *R*_2_* relaxivity; molar susceptibility, respectively.

To quantify measurement precision, the standard deviation of the ROI measurement (SD_ROI_) was reported as median (range) for concentrations 1 to 5 of each material group. We also reported test–retest repeatability of mean ROI measurement using a single‐score coefficient of reliability (ICC) calculated using a two‐way ANOVA model with absolute agreement (‘A − 1’) [63, 64].

To determine material lifespan, we performed a *t*‐test to detect a significant difference between baseline *χ*_mol_ values to timepoint *χ*_mol_ value; the first timepoint at which there was a significant change was determined to be the material lifespan. We also performed a *t*‐test to detect a possible correlation between time (*t* = 0, 9, 24 months) and timepoint *χ*_mol_ values.

Bland–Altman (BA) analysis for repeated measurements per subject (phantom) [65] was applied to evaluate agreement between 3 and 7 T scanners. We performed the following BA analyses: (1) *χ*_3T_ versus *χ*_7T_ for ferritin, CaCl_2_ and CaCO_3_, (2) *M*_3T_ versus *M*_7T_ (where *M* = *χ*·*B*_0_/*μ*_0_) for USPIO, (3) (*R*_2_*/*B*_0_)_3T_ versus (*R*_2_*/*B*_0_)_7T_ for CaCO_3_ and ferritin, and (4) (*R*_2_*)_3T_ versus (*R*_2_*)_7T_ for USPIO and CaCl_2_.

## 3 Results

**FIGURE 2:**
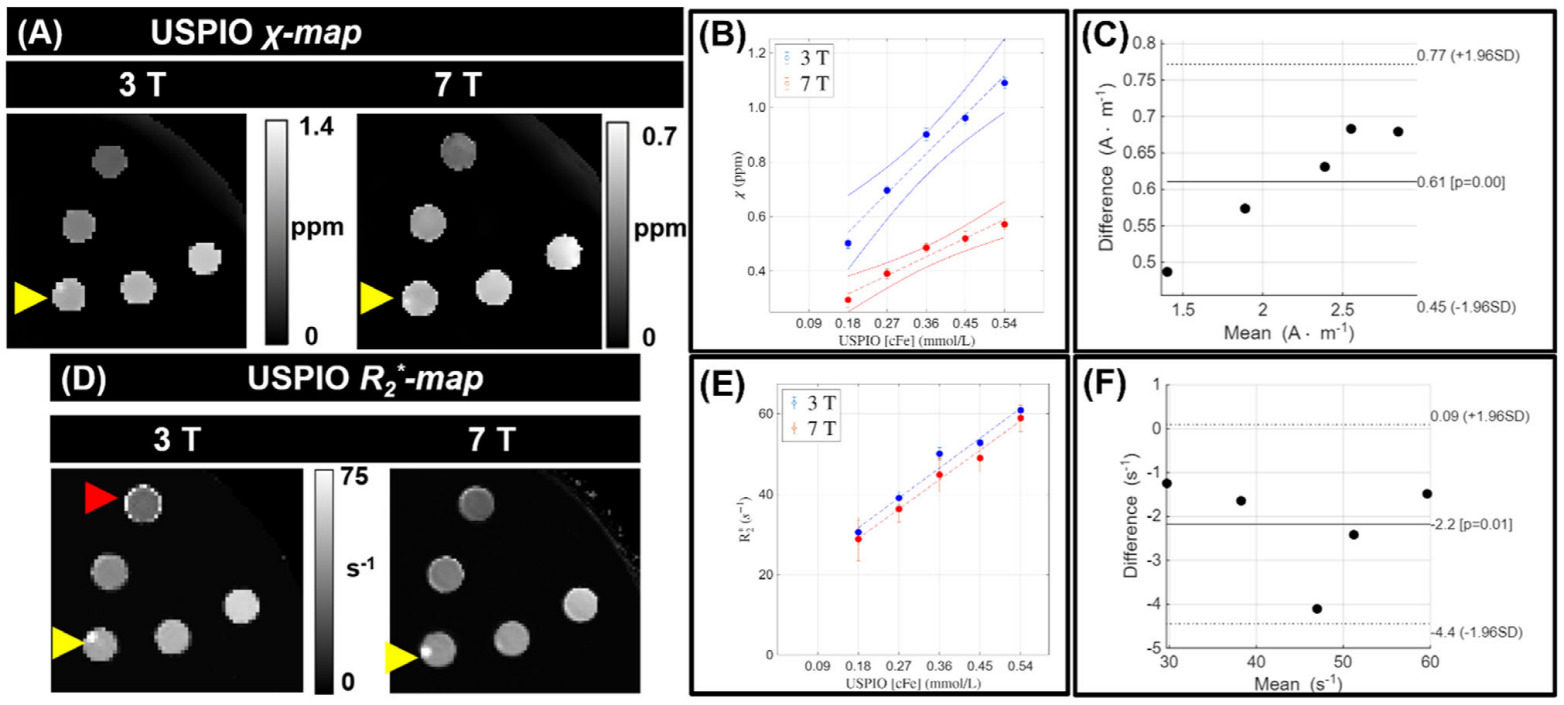
USPIO observation regions within maps of *χ* (A) and *R*_2_* (D). The windowing of the *χ* map at 7 T is set to half that of 3 T. The yellow arrowhead indicates a clump, manifesting as bright spot on both χ and *R*_2_* maps. The red arrowhead indicates a *R*_2_* fitting error at the vial edge. (B, E) Scatter plots of (B) *χ* and *R*_2_* (E) as a function of USPIO concentration. (C, F) Bland–Altman plots of (C) *M*_3T_ versus *M*_7T_ and (F) (*R*_2_*)_3T_ versus (*R*_2_*)_7T_.

**FIGURE 3:**
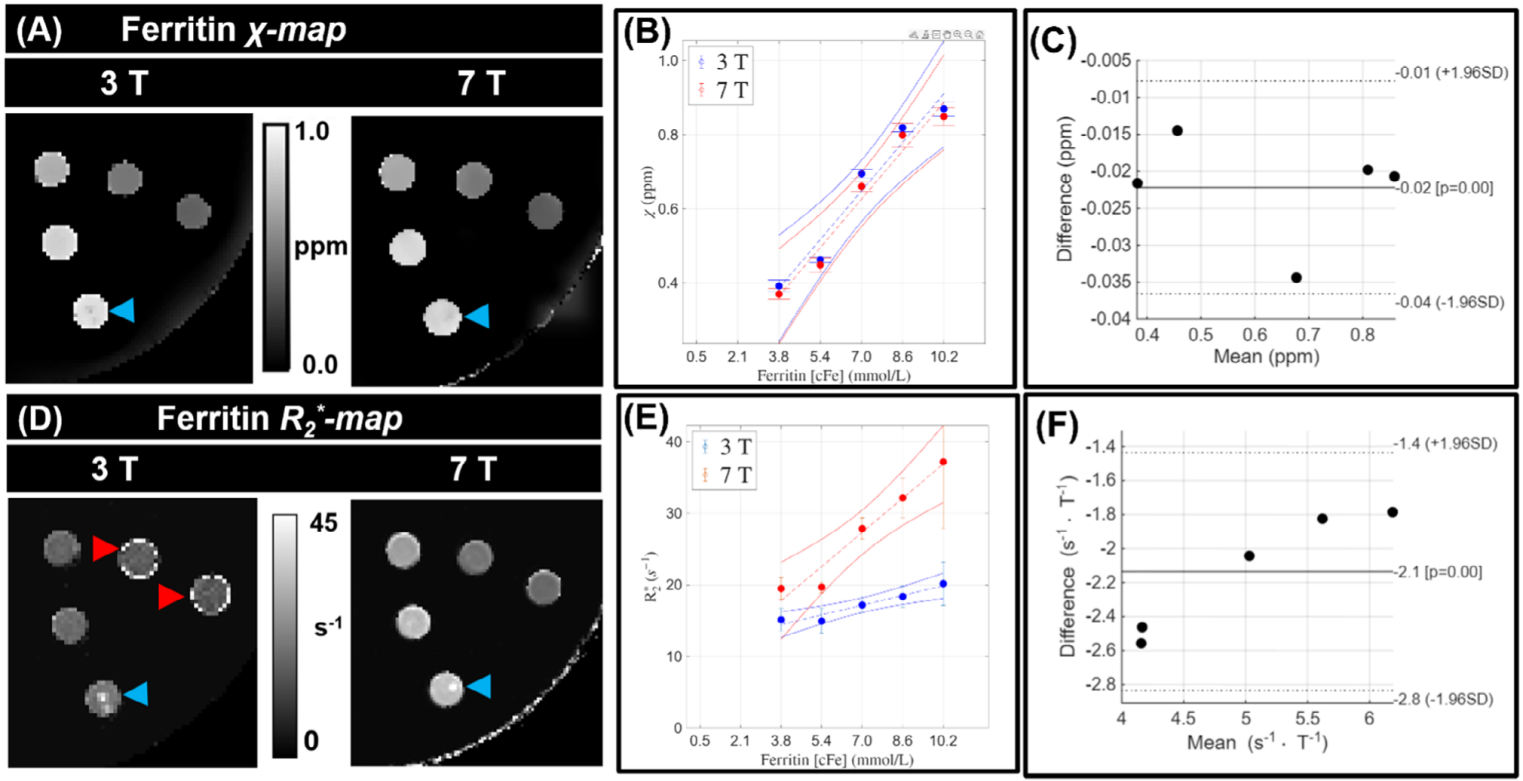
Ferritin observation regions within maps of *χ* (A) and *R*_2_* (D). At 10.2 mmol/L, a clump is marked with a blue arrowhead, manifesting as dark on *χ* (diamagnetic) and bright on *R*_2_*. The red arrowheads indicate a *R*_2_* fitting error at the vial edge. (B, E) Scatter plots of (B) *χ* and (E) *R*_2_* as a function of ferritin concentration. (C, F) Bland–Altman plots of (C) *χ*_3T_ versus *χ*_7T_ and (F) (*R*_2_*/*B*_0_)_3T_ versus (*R*_2_*/*B*_0_)_7T_.

**FIGURE 4:**
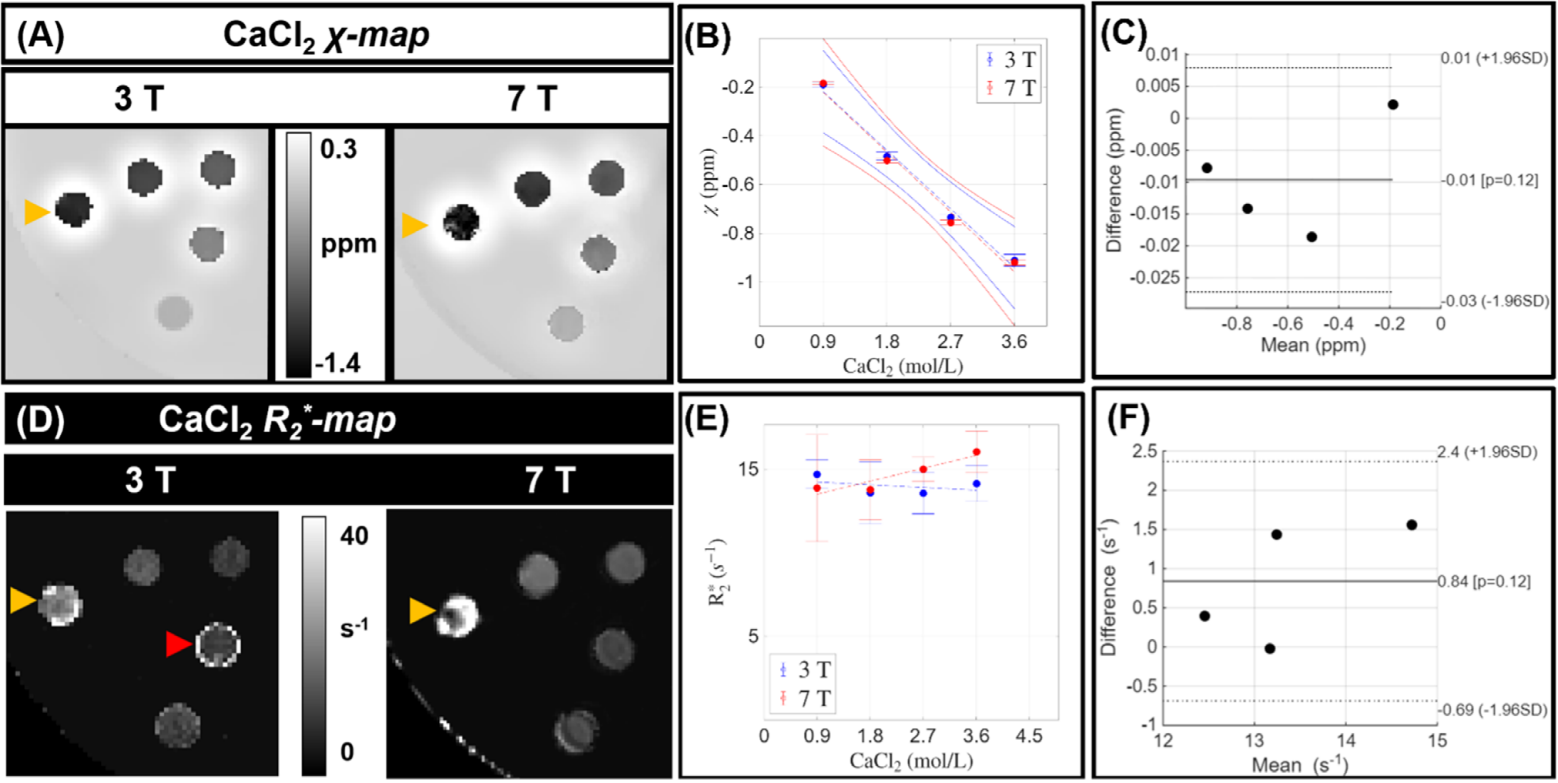
Calcium chloride (CaCl_2_) observation regions within maps of *χ* (A) and *R*_2_* (D). The voxels corresponding to CaCl_2_ 4.5 mol/L are heterogeneous, as indicated with an orange arrowhead on each *χ* map. The red arrowhead indicates a *R*_2_* fitting error at the vial edge. (B, E) Scatter plots of *χ* (B) and *R*_2_* (E) as a function of concentration of CaCl_2_. (C, F) Bland–Altman plots of (C) *χ*_3T_ versus *χ*_7T_ and (F) (*R*_2_*)_3T_ versus (*R*_2_*)_7T_.

**FIGURE 5:**
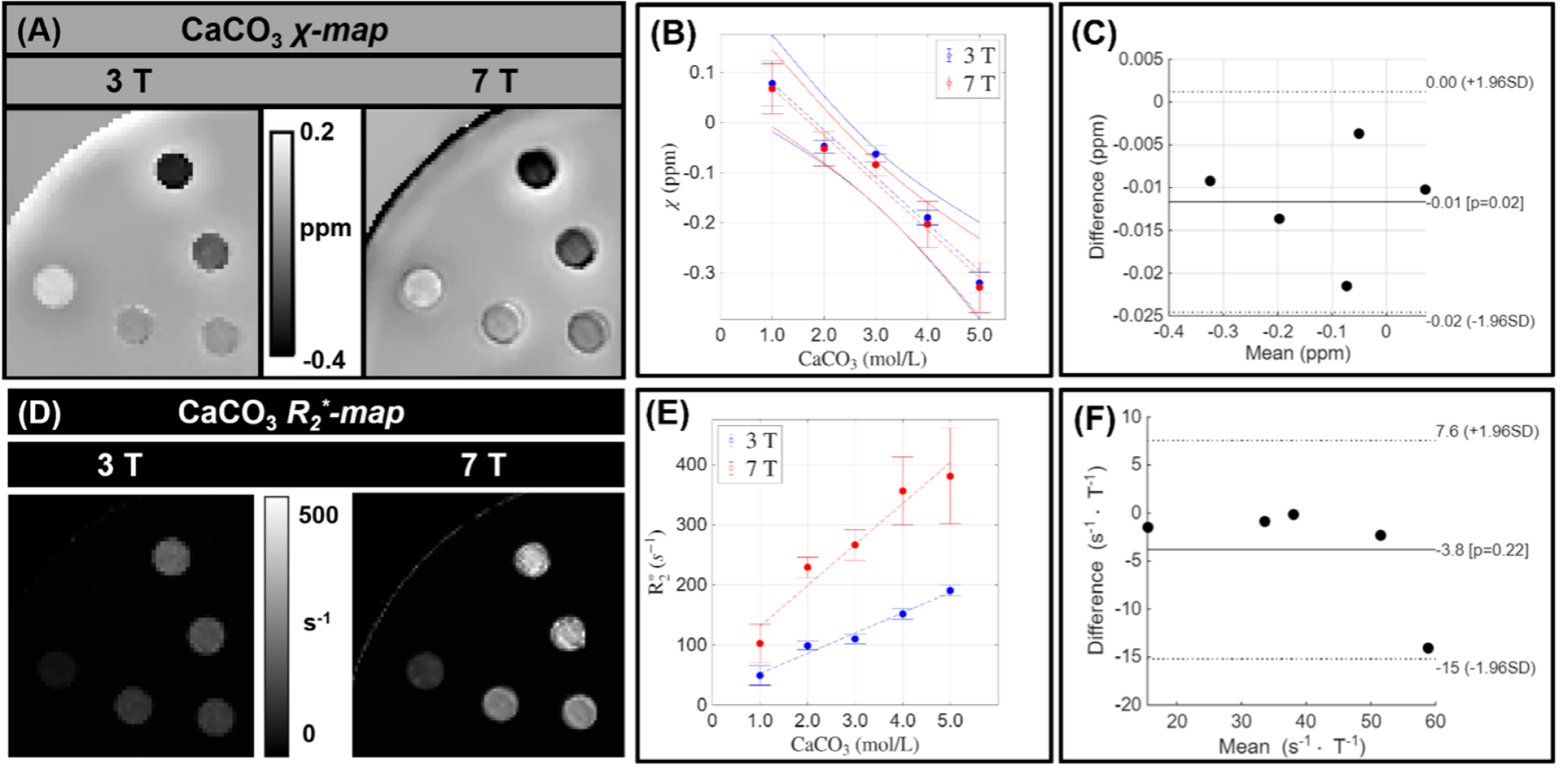
Calcium carbonate (CaCO_3_) observation regions within maps of *χ* (A) and *R*_2_* (D). (B, E) Scatter plots of *χ* (B) and *R*_2_* (E) as a function of concentration of CaCO_3_. (C, F) Bland–Altman plots of (C) *χ*_3T_ versus *χ*_7T_ and (F) (*R*_2_*/*B*_0_)_3T_ versus (*R*_2_*/*B*_0_)_7T_.

Maps and plots of susceptibility and *R*_2_* for the four used materials and five concentrations are shown in Figures 2, 3, 4, 5. On the scatter plots, the fitted regression is dotted, and, where given, the 95% confidence intervals are solid. The vertical error bars are the standard deviation of the ROI measurement. The Bland‐Altman mean difference (bias) line is solid, and the limits‐of‐agreement are dotted.

### 3.1 Dopant Clump Analysis and Other Confounds

At 3 T, the volume of elevated *R*_2_* observed for ferritin (3.49 mm^3^) was roughly 6% larger that of USPIO (3.27 mm^3^). At 7 T, the mean volume for ferritin (13.01 mm^3^) was roughly twice that of USPIO (6.40 mm^3^). A large clump with positive susceptibility is shown for USPIO (see Figure 2) and a clump with negative susceptibility is shown for ferritin (see Figure 3).

For CaCl_2_ 4.5 mol/L, concentrations near this level approach the solubility limit (∼4.0 mol/L) [33], which resulted in visible particulate aggregations in our samples. CaCl_2_ 4.5 mol/L was problematic at both field strengths and thus excluded from statistical analysis. For CaCl_2_, the volumes of elevated *R*_2_* at 3 T (1.46 mm^3^) were roughly 4 times less than those at 7 T (5.59 mm^3^), as indicated in Table S2.

Referring to Figure 5F, CaCO_3_ 1.0 to 4.0 mol/L showed differences between −0.2 to −2.4 s^−1^ T^−1^, but CaCO_3_ 5.0 mol/L showed a difference of −14.1 s^−1^ T^−1^. For CaCO_3_, the clump sizes at 3 T (1.27 mm^3^) were roughly seven times less than the clump sizes at 7 T (10.40 mm^3^), as indicated in Table S2.

### 3.2 Validation of Echo Time Selection for CaCO_3_ ROIs

Table S5 compared the linearity (CaCO_3_ concentration as a function of susceptibility) derived with all echo times against the linearity derived with later echo times excluded. At 3 T, an excellent linearity (*R* ^2^ = 0.94) was observed in each case. At 7 T, using all echo times leads to a poor linearity (*R* ^2^ = 0.19), and excluding later echo times leads to an excellent linearity (*R* ^2^ = 0.96).

### 3.3 Linear Regression (*c*_mol_ vs. *χ*)

The linearity was excellent, *R* ^2^ > 0.9, for all four materials and field strengths (see Table 2). For USPIO, *χ*_mol_ at 3 T (1.67 ppm mmol^−1^ L) was roughly 2.3 times greater than its *χ*_mol_ at 7 T (0.74 ppm mmol^−1^ L). For ferritin, *χ*_mol_ at 3 and 7 T were equal (0.0813 ppm mmol^−1^ L). For CaCl_2_, *χ*_mol_ at 3 T (0.268 ppm mol^−1^ L) was roughly 1% less than its *χ*_mol_ at 7 T (0.271 ppm mol^−1^ L). For CaCO_3_, *χ*_mol_ at 3 T (0.0952 ppm mol^−1^ L) was also roughly 1% less than its *χ*_mol_ at 7 T (0.0953 ppm mol^−1^ L).

**TABLE 2:** Linear fitting coefficients (*c*_mol_ vs. *χ*) and linearity (*R* ^2^) at 3, 7 T, respectively. Coefficients were reported with standard error.

	USPIO	Ferritin	CaCl_2_	CaCO_3_
*χ*_mol_ (ppm L mmol^−1^)	1.67 ± 0.24; 0.74 ± 0.09	10^−2^ × (8.13 ± 1.35); 10^−2^ × (8.13 ± 1.19)	10^−4^ × (−2.68 ± 0.24); 10^−4^ × (−2.71 ± 0.37)	10^−5^ × (−9.52 ± 1.44); 10^−5^ × (−9.53 ± 1.18)
*χ*_0_ (ppb)	253 ± 61; 188 ± 28	80 ± 81; 53 ± 91	25 ± 80; 19 ± 91	190 ± 43; 165 ± 39
*R* ^2^ (*c*_mol_ vs. *χ*)	0.95; 0.95	0.95; 0.94	0.98; 0.97	0.94; 0.96

### 3.4 Linear Regression (*c*_mol_ vs. *R*_2_ *)

The linearity was poor (*R* ^2^ < 0.9) for CaCl_2_ and excellent (*R* ^2^ > 0.9) for CaCO_3_, USPIO and ferritin (see Table S3). For USPIO, *R*_2_*_mol_ at 7 T (79.5 s^−1^ mmol^−1^ L) was roughly 6% less than at 3 T (84.2 s^−1^ mmol^−1^ L). For ferritin, *R*_2_*_mol_ at 7 T (0.77 s^−1^ mmol^−1^ L) was roughly four times less than at 3 T (2.78 s^−1^ mmol^−1^ L). Since the linearity of CaCl_2_ was poor, we report its mean ± standard deviation as 14.0 ± 0.5 s^−1^; 14.7 ± 1.1 s^−1^ at 3, 7 T, respectively. For CaCO_3_, *R*_2_*_mol_ at 7 T (86.9 s^−1^ mol^−1^ L) was roughly 2.6 times greater than at 3 T (33.7 s^−1^ mol^−1^ L).

### 3.5 Precision of Susceptibility Measurements

The standard deviation of the ROI measurement (SD_ROI_) was expressed as median (minimum‐maximum) for concentrations 1 to 5 of each material group (see Table 3). For USPIO, the median at 7 T (21.5 ppb) was 11% greater than the median SD_ROI_ at 3 T (19.3 ppb). For ferritin, the median SD_ROI_ at 7 T (18.9 ppb) was 56% greater than the median SD_ROI_ at 3 T (12.1 ppb). For CaCl_2_, the median SD_ROI_ at 7 T (32.7 ppb) was three times greater than the median SD_ROI_ at 3 T (17.3 ppb). Similarly, for CaCO_3_ the median SD_ROI_ at 7 T (45.7 ppb) was roughly three times greater than the median SD_ROI_ at 3 T (16.4 ppb). The test–retest repeatability was ‘excellent’ (ICC > 0.90) for each material.

**TABLE 3:** Precision and lifespan of *χ* measurements for concentrations 1 to 5 of each material group.

	USPIO	Ferritin	CaCl_2_	CaCO_3_
SD_ROI_ (ppb)	19.3 (14.9–23.5); 21.5 (16.9–27.2)	12.1 (8.5–19.6); 18.9 (14.0–32.6)	10.9 (6.3–15.4); 32.7 (24.0–52.5)	16.4 (12.6–45.3); 45.7 (21.3–49.9)
ICC, test–retest	0.99–1.00; 0.99–1.00	0.99–1.00; 1.00–1.00	0.99–1.00; 1.00–1.00	1.00–1.00; 1.00–1.00
Material lifespan	≤ 24 months (*p* = 0.05 at 3 T)	> 24 months (no significant change observed)	≤ 9 months (*p* = 0.04 at 7 T)	> 15 months (no significant change observed)

*Note:* Besides material lifespan, values given at 3, 7 T, respectively. Standard deviation of the ROI measurement (SD_ROI_) was reported as median (min–max). Coefficient of reliability (ICC) was reported as: ICC lower interval to ICC upper interval. Material lifespan was based on *t*‐tests comparing baseline *χ*_mol_ values to *χ*_mol_ value at various timepoints.

### 3.6 Material Lifespan

Comparing the molar susceptibility measurements from *t*_0_ to *t*_1_ showed nil significant change for USPIO (*p* = 0.32; *p* = 0.06 at 3, 7 T), ferritin (*p* = 0.55; *p* = 0.11 at 3, 7 T), CaCl_2_ at 3 T (*p* = 0.71), but there was a significant change for CaCl_2_ at 7 T (*p* = 0.04). Comparing the molar susceptibility measurements from *t*_0_ to *t*_2_ showed a significant change for USPIO at 3 T (*p* = 0.05) but not at 7 T (*p* = 0.10), and no significant change for ferritin (*p* = 0.24; *p* = 0.32 at 3, 7 T). Comparing the molar susceptibility measurements from *t*_1_ to *t*_2_ showed no significant change for CaCO_3_ (*p* = 0.38; *p* = 0.12 at 3, 7 T). There was no significant linear correlation between time (*t*_month_ = [0,9,24]) and molar susceptibility (*χ*_mol_) for USPIO (*p* = 0.38; *p* = 0.82 at 3, 7 T) and ferritin (*p* = 0.28; *p* = 0.70 at 3, 7 T).

### 3.7 Cross‐Field‐Strength Agreement

The results of the Bland–Altman analysis were summarized in Table S4. *χ*_3T_ versus *χ*_7T_ indicated a bias of −0.02 ppm (*p* = 0.00) for ferritin, −0.01 ppm (*p* = 0.12) for CaCl_2_, and −0.01 ppm (*p* = 0.02) for CaCO_3_. *M*_3T_ versus *M*_7T_ showed a bias of 0.61 A/m (*p* = 0.00) for USPIO. (*R*_2_*/*B*_0_)_3T_ versus (*R*_2_*/*B*_0_)_7T_ revealed a bias of −2.1 s^−1^ T^−1^ (*p* = 0.00) for ferritin and −3.8 s^−1^ T^−1^ (*p* = 0.22) for CaCO_3_. (*R*_2_)_3T_ versus (*R*_2_*)_7T_ showed a bias of −2.2 s^−1^ (*p* = 0.01) for USPIO, and 0.84 s^−1^ (*p* = 0.12) for CaCl_2_.

## 4 Discussion

### 4.1 Acquisition and Processing

This study used MR acquisition parameters that were mostly consistent with those recommended by experts at 3 T [66]. One limitation of this study was the increased bandwidth of 1000 Hz/pix at 3 T, which increased noise within the image, and enhanced Gibbs (truncation) artifacts, but these Gibbs artifacts were corrected during pre‐processing (as described in Section 2.3).

### 4.2 Dopant Clumping

We observed minor deviations in SNR and local field surrounding the dopant clumps. The mean number of elevated *R*_2_* voxels was small (less than 3.0% for each material group, as indicated in Table S2). Errors associated with dopant clumps were mitigated by modulating the weighting map using information from a simulated mono‐exponential signal (as described in Section 2.5). Error propagation is reduced through the use of information from magnitude images to accurately regularize the susceptibility gradients in the MEDI algorithm [61]. Moreover, the MEDI algorithm uses a non‐linear data fidelity term, which better handles dipole incompatible fields than the linear data fidelity variant [47].

### 4.3 CaCO_3_ Concentrations With Acceptable Signal Level

A critical issue with using concentrated CaCO_3_ was that later echo times had SNR below our empirically determined threshold of 10:1; due to the short *T*_2_* of the concentrated CaCO_3_. For CaCO_3_, the median SD_ROI_ at 3 T (16.4 ppb) was roughly three times less than the median SD_ROI_ at 7 T (45.7 ppb)—caused by extremely short *T*_2_* values at 7 T. It is desirable to identify a CaCO_3_ concentration limit without having to remove later echo times, which can be identified by comparing *T*_2_* to clinically relevant echo times [66]: 5 to 29 ms at 3 T and 4 to 20 ms at 7 T. Optimizing SNR in phase images typically requires a TE value between *T*_2_*/2 and *T*_2_* to sustain an acceptable signal level [67]. If three echo times are used for QSM reconstruction (and assuming first echo time and echo spacing are equal), then we identify a CaCO_3_ concentration limit by setting *T*_2_*/3 to equal the shortest echo time of the clinically relevant echo times [66]; the CaCO_3_ concentration limit is then between 1.0 mol/L (*T*_2_*/3 = 7.1 ms) to 2.0 mol/L (*T*_2_*/3 = 3.4 ms) at 3 T, and less than 1.0 mol/L (*T*_2_*/3 = 3.2 ms) at 7 T. If we define an “acceptable signal level” using our empirically determined threshold of 10:1 (see Table S1), then we return the same result.

### 4.4 CaCO_3_ Particle Size

The *T*_2_* relaxation induced by insoluble CaCO_3_ is expected to depend strongly on particle size via the transition between static and dynamic dephasing regimes, whereas the apparent susceptibility is comparatively insensitive to particle size. A limitation of the current work was the absence of CaCO_3_ particle size measurement. Emmerich et al. explicitly selected CaCO_3_ particles with median diameters of 8.7 μm to ensure the assumptions of the static dephasing regime were satisfied, which allowed *R*_2_*/*B*_0_ to be used as a quantitative proxy for susceptibility [30].

### 4.5 CaCl_2_ Shading Artifacts

Changes in conductivity can lead to RF nonuniformity and RF shielding and consequently ‘shading’ artifacts. In the current study, the shading artifacts were caused by the high conductivity of concentrated (0.9 mol/L or larger) CaCl_2_ solutions (approximately 106–183 mS cm^−1^) [33]. Such high conductivity was sufficient to perturb the transmit RF field at 7 T leading to pronounced shading artifacts adjacent to CaCl_2_ vials, as indicated in Figure S2, but which were not observed at 3 T.

### 4.6 Ferritin and CaCO_3_ Material Stability

A key aim within the quantitative MRI community is the development of phantoms that can assist system stability [68, 69]. This requires materials that are stable within minutes of repeated measurements (test–retest measurements), as well as within months (longitudinal measurements). In terms of material lifespan, ferritin and CaCO_3_ each were stable over the measured timeframes (9 and 24 months for ferritin; 15 months for CaCO_3_). Ferritin and CaCO_3_ could therefore be used within susceptibility phantoms to support 3/7 T harmonization studies, using our measurement data as a reference when constructing such a phantom.

### 4.7 Crossfield‐Strength Agreement

The motivation behind using an ultra‐high‐field scanner is to acquire images at increased imaging resolution relative to those used in clinical scanners without trading off SNR and imaging time. Optimizing and standardizing QSM protocols used for both clinical and ultra‐high‐field scanners are essential in making susceptibility a robust biomarker [70]. Initiatives such as the German Ultrahigh Field Imaging network traveling heads study [71, 72] and the United Kingdom 7 T study [73] are steps towards applying quantitative MRI at ultra‐high‐field. In the current work, Bland–Altman analysis between *χ*_3T_ and *χ*_7T_ (summarized in Table S4) indicated a bias of −0.02 ppm (*p* = 0.00) for ferritin, −0.01 ppm (*p* = 0.12) for CaCl_2_ and −0.01 ppm (*p* = 0.02) for CaCO_3_. Curiously, the agreement between *χ*_3T_ and *χ*_7T_ was best at concentrations 1 and 5 (vials positioned closest to the edge). This is likely due to those vials being closer to the coils, therefore, receiving a higher SNR than concentrations 2 to 4 (vials positioned closest to the center). An important property of CaCO_3_ is that the quotient of *R*_2_* and field strength, *R*_2_*/*B*_0_, is invariant with field strength [31]. Bland–Altman analysis between (*R*_2_*/*B*_0_)_3T_ and (*R*_2_*/*B*_0_)_7T_ (summarized in Table S4) revealed a bias of −3.8 s^−1^ T^−1^ (*p* = 0.22) for CaCO_3_. The bias might be reduced by using lower CaCO_3_ concentrations than those used in the current work.

### 4.8 Comparison to Reference Values

For USPIO formulations such as MoldayION and Ferumoxytol, the relationship between magnetic field strength and magnetization is nonlinear [5] (see Figure S4). Since the USPIO saturation field is around 1T [74], the USPIO magnetization is saturated at 3 and 7 T. In comparison, most materials observed in vivo MRI (e.g., air, water and tissue) have a linear response (see Figure S4). Liu et al. reported molar susceptibility values of 1.79 and 0.74 ppm L mmol^−1^ at 3 and 7 T, respectively [4]. These values fall within the range of USPIO molar susceptibility measured in the current study, 1.67 ± 0.24 and 0.74 ± 0.09 ppm L mmol^−1^ at 3 and 7 T, respectively. The molar susceptibility of ferritin can be derived by Curie's Law [4], with effective Curie magnetic moment equal to 3.78 Bohr Magnetons [75, 76] leading to a molar susceptibility value of 76.6 ppm L mol^−1^ at 293 K. This value falls within the range of ferritin molar susceptibility measured in the current study, 81 ± 14 and 81 ± 12 ppm L mol^−1^ at 3 and 7 T. Experimental validation studies indicate the susceptibility value of any paramagnetic material may deviate with temperature [4], and the internal temperature of the phantom bore frequently deviates from the ambient temperature of the scanner room [68]. Future studies would therefore benefit from using integrated temperature monitoring and/or control [77, 78].

## 5 Conclusion

This research completed the following quantitative analysis of phantom materials: signal‐to‐noise ratio, dopant clump analysis, test–retest repeatability, crossfield‐strength agreement, and material lifespan. We recommend using ferritin as a paramagnetic dopant. Further research is required to identify a diamagnetic dopant with a lower electrical conductivity and a lower ratio of *R*_2_*/*B*_0_ to *χ*.

## Funding

This work was supported by Australian Research Council, IC170100035.

## Conflicts of Interest

Kieran O'Brien and Jin Jin are employees of Siemens Healthineers in Australia. The other authors declare no conflicts of interest.

## Supporting information


**Figure S1:** Computation of reference mask (M_R_ in Equation 4): (A) *R*_2_* thresholding at 5 s^−1^ to separate the vials from the background fluid, indicating *R*_2_* errors with red arrows. (B) Morphological closing (to fill in small holes within the background fluid, while preserving the larger holes of the vials). (C) Morphological erosion (since cylinders produce an external field affected by the object's susceptibility, we must exclude this external field when referencing).
**Figure S2:** RF nonuniformity observed as ‘shading’ artifacts are prominent at 7 T at the borders of the vials depending on the high conductivity of concentrated CaCl_2_ on (C) magnitude and (D) phase images. These artifacts are not visible on (A) magnitude and (B) phase images at 3 T. The transmit RF field (B_1_^+^) map at 7 T is shown in (E).
**Figure S3:** Sagittal view of 3 T magnitude image used in MEDI processing (A) and susceptibility maps reconstructed with (B) c_∇_ = 0.1, (C) c_∇_ = 0.5, and (D) c_∇_ = 0.9. The vial chosen here for demonstration was ferritin 10.2 mmol/L which contained four clumps embedded in ferritin‐agarose mixture (presenting as four dark clusters). Susceptibility maps with c_∇_ = 0.5 in (C) preserve morphological detail, while using c_∇_ = 0.1 (B) shows blurred morphological detail and reduced contrast. Using c_∇_ = 0.9 (D) shows increased inhomogeneity.
**Figure S4:** Illustration of specific magnetization and molar susceptibility curves for USPIO and ferritin. Computed using literature values: specific magnetization (per Fe_3_O_4_) for Ferumoxytol (23.7 to 25.2 A m^2^ kg^−1^ between 2 and 7 T [0;[1, 2]) and molar susceptibility for ferritin (*χ*_mol_ = 0.0766 ppm L mmol^−1^ [3]) at 293 K. USPIO produce a nonlinear response in magnetization, *M* = *M*_s_·L(*α*) [4], in which *M*_s_ is the nanoparticle's saturation magnetization, *L*(*α*) is the Langevin function, and *α* is a function of the nanoparticle's magnetic moment, sample temperature and the applied magnetic field (*B*_0_) [4]. In comparison, ferritin shows a linear response in magnetization with changes in applied field. Magnetic susceptibility was related to magnetization by *χ* = *M μ*_0_/*B*_0_ in which *μ*_0_ denotes vacuum magnetic permeability.
**Table S1:** SNR at each TE for each material concentration, at (a) 3 T, and (b) 7 T, respectively. The SNR ranges were defined as follows; high SNR (in green): SNR ≥ 50:1, intermediate SNR (in yellow): 20 ≤ SNR < 50, borderline (in orange): 10 ≤ SNR < 20, low (in red): < 10.
**Table S2:** Volume of elevated *R*_2_* and voxels of elevated *R*_2_* at 3, 7 T. All values were reported as mean ± standard deviation.
**Table S3:** Linear fitting coefficients (*c*_mol_ vs. *R*_2_*) and linearity (*R* ^2^) at 3, 7 T, respectively. Coefficients were reported with standard error.
**Table S4:** Bland–Altman analysis (bias ± limit‐of‐agreement). The results of a *t*‐test were shown using zero bias as the null hypothesis. **M_3T_ versus *M*_7T_ was assessed for USPIO, and (*R*_2_*/*B*_0_)_3T_ versus (*R*_2_*/*B*_0_)_7T_ was assessed for CaCO_3_ and ferritin.
**Table S5:** Susceptibility value (in ppb units) at CaCO_3_ ROIs derived from susceptibility maps reconstructed with all echo times (*χ*_all‐TE_) and with later echo times excluded (*χ*_excl‐TE_). Coefficient of determination (*R* ^2^) was used to evaluate the goodness‐of‐fit between molar concentration and susceptibility.

## Data Availability

We facilitate the reproducibility of this study and the phantom by providing imaging data (10.5281/zenodo.16295918) and scripts (https://github.com/paddyhooper93/Thesis, commit hash 5ea6db1).
