# Supplementary material for: Evaluation of Phantom Doping Materials in Quantitative Susceptibility Mapping
Source: Magn Reson Med. 2026 Jun 25;96(4):1545–56. doi: 10.1002/mrm.70427 (PMC13419044; doi:10.1002/mrm.70427)
Supplement: Supplementary file 1 — Figure S1: Computation of reference mask (MR in Equation 4): (A) R 2* thresholding at 5 s−1 to separate the vials from the background fluid, indicating R 2* errors with red arrows. (B) Morphological closing (to fill in small holes within the background fluid, while preserving the larger holes of the vials). (C) Morphological erosion (since cylinders produce an external field affected by the object's susceptibility, we must exclude this external field when referencing). Figure S2: RF nonuniformity observed as ‘shading’ artifacts are prominent at 7 T at the borders of the vials depending on the high conductivity of concentrated CaCl2 on (C) magnitude and (D) phase images. These artifacts are not visible on (A) magnitude and (B) phase images at 3 T. The transmit RF field (B1 +) map at 7 T is shown in (E). Figure S3: Sagittal view of 3 T magnitude image used in MEDI processing (A) and susceptibility maps reconstructed with (B) c∇ = 0.1, (C) c∇ = 0.5, and (D) c∇ = 0.9. The vial chosen here for demonstration was ferritin 10.2 mmol/L which contained four clumps embedded in ferritin‐agarose mixture (presenting as four dark clusters). Susceptibility maps with c∇ = 0.5 in (C) preserve morphological detail, while using c∇ = 0.1 (B) shows blurred morphological detail and reduced contrast. Using c∇ = 0.9 (D) shows increased inhomogeneity. Figure S4: Illustration of specific magnetization and molar susceptibility curves for USPIO and ferritin. Computed using literature values: specific magnetization (per Fe3O4) for Ferumoxytol (23.7 to 25.2 A m2 kg−1 between 2 and 7 T [0;[1, 2]) and molar susceptibility for ferritin (χ mol = 0.0766 ppm L mmol−1 [3]) at 293 K. USPIO produce a nonlinear response in magnetization, M = M s·L(α) [4], in which M s is the nanoparticle's saturation magnetization, L(α) is the Langevin function, and α is a function of the nanoparticle's magnetic moment, sample temperature and the applied magnetic field (B 0) [4]. In comparison, ferritin shows a linear resp [file MRM-96-1545-s001.docx]

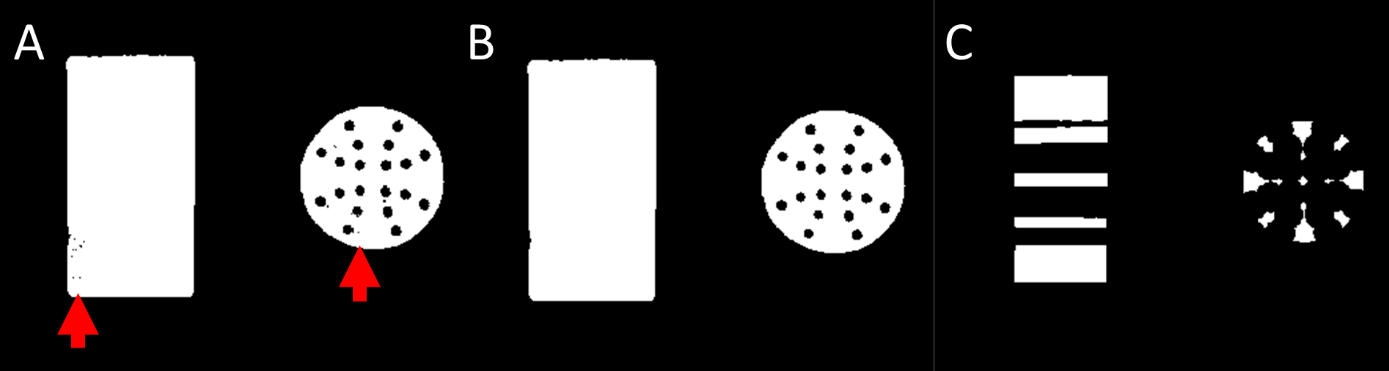


Figure S1: Computation of reference mask (M_R_ in Equation 4): (A) R_2_^*^ thresholding at 5 s^−1^ to separate the vials from the background fluid, indicating R_2_^*^ errors with red arrows. (B) Morphological closing (to fill in small holes within the background fluid, while preserving the larger holes of the vials). (C) Morphological erosion (since cylinders produce an external field affected by the object’s susceptibility, we must exclude this external field when referencing).


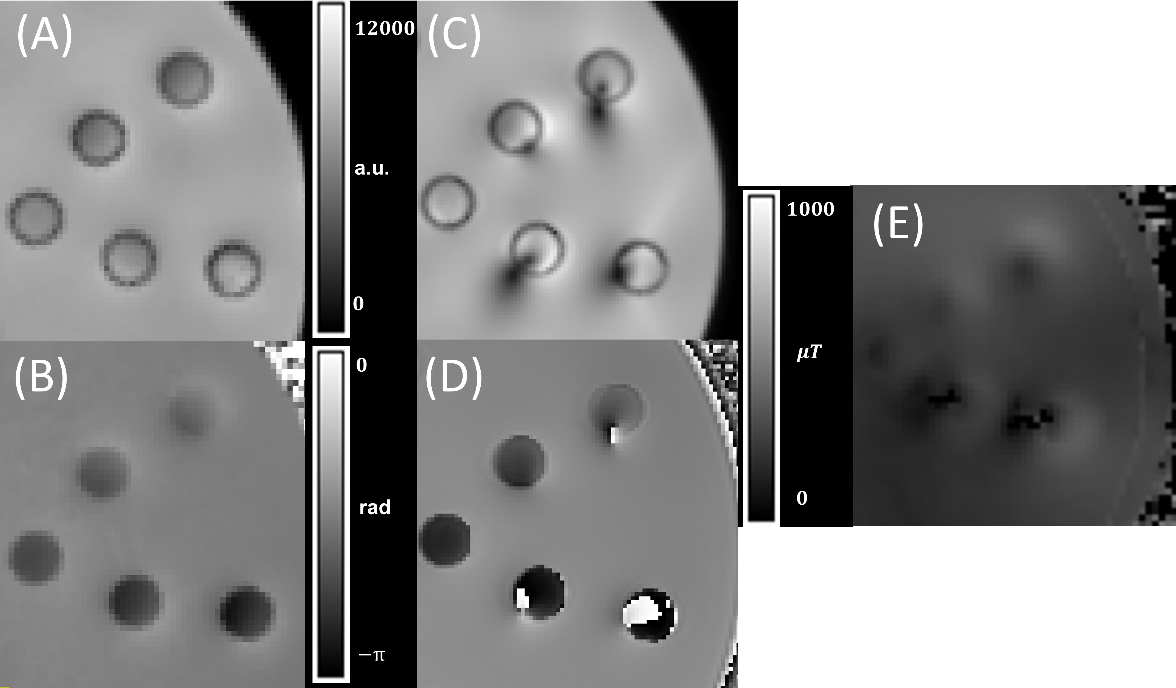


Figure S2: RF nonuniformity observed as ‘shading’ artifacts are prominent at 7 T at the borders of the vials depending on the high conductivity of concentrated CaCl_2_ on (C) magnitude and (D) phase images. These artifacts are not visible on (A) magnitude and (B) phase images at 3 T. The transmit RF field (*B*_1_^+^) map at 7 T is shown in (E).


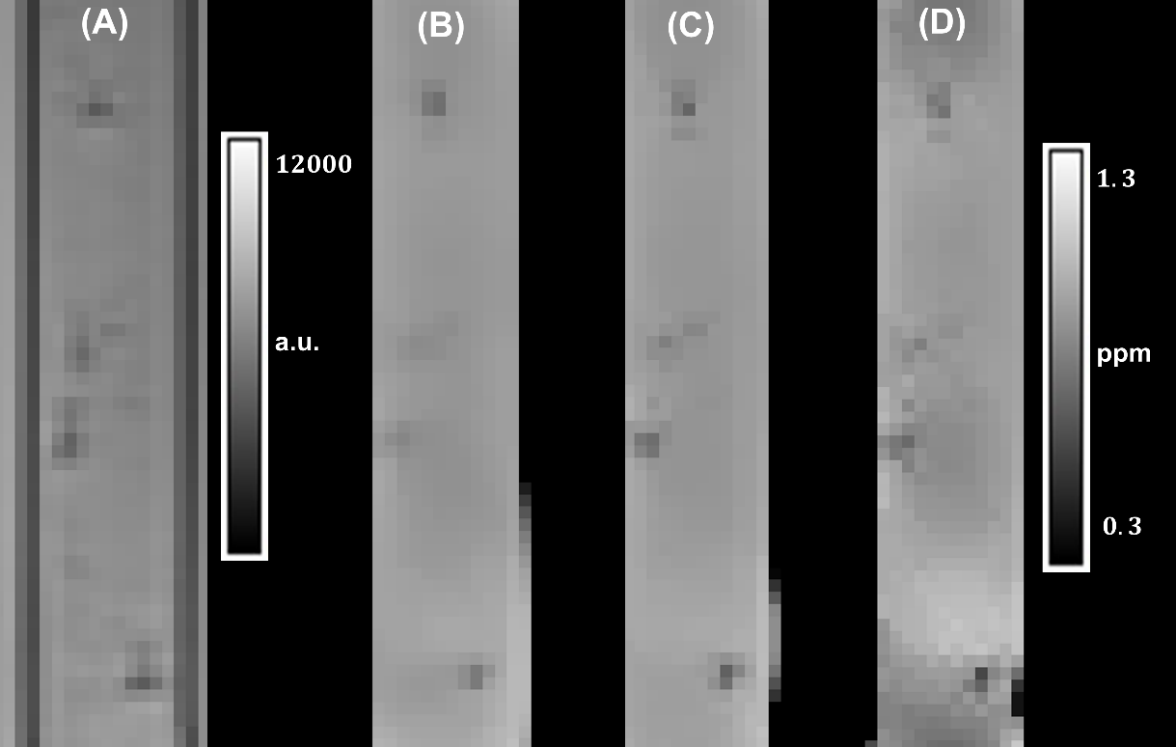


Figure S3: Sagittal view of 3T magnitude image used in MEDI processing (A) and susceptibility maps reconstructed with (B) c_∇_=0.1, (C) c_∇_=0.5, and (D) c_∇_=0.9. The vial chosen here for demonstration was ferritin 10.2 mmol/L which contained 4 clumps embedded in ferritin-agarose mixture (presenting as 4 dark clusters). Susceptibility maps with c_∇_=0.5 in (C) preserve morphological detail, while using c_∇_=0.1 (B) shows blurred morphological detail and reduced contrast. Using c_∇_=0.9 (D) shows increased inhomogeneity.


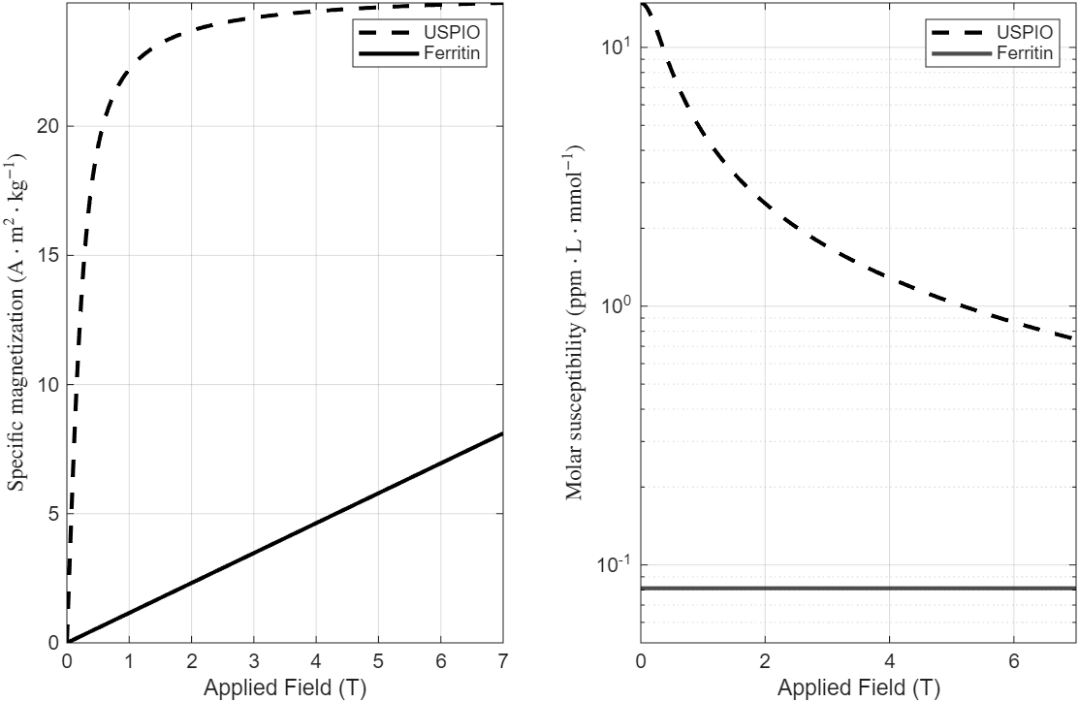


Figure S4: Illustration of specific magnetization and molar susceptibility curves for USPIO and ferritin. Computed using literature values: specific magnetization (per Fe_3_O_4_) for Ferumoxytol (23.7 to 25.2 A∙m^2^∙kg^-1^ between 2T and 7T^1,2^) and molar susceptibility for ferritin (χ_mol_=0.0766 ppm·L·mmol^−1^ ^3^) at 293 K. USPIO produce a nonlinear response in magnetization, M = M_s_∙L(α)^4^, in which M_s_ is the nanoparticle’s saturation magnetization, L(α) is the Langevin function, and α is a function of the nanoparticle’s magnetic moment, sample temperature and the applied magnetic field (B_0_)^4^. In comparison, ferritin shows a linear response in magnetization with changes in applied field. Magnetic susceptibility was related to magnetization by χ = M∙μ_0_/B_0_ in which μ_0_ denotes vacuum magnetic permeability.

| Table S1 (a) | | TE1: 1.87 | TE2: 3.74 | TE3: 5.61 | TE4: 7.48 | TE5: 9.35 | TE6: 11.22 | TE7: 13.09 | TE8: 14.96 | TE9: 16.83 | TE10: 18.7 | TE11: 20.57 | TE12: 22.44 |
| --- | --- | --- | --- | --- | --- | --- | --- | --- | --- | --- | --- | --- | --- |
| Fe mmol/L | |  | | | | | | | | | | | |
| USPIO | 0.224 | 137 | 125 | 119 | 112 | 106 | 99 | 97 | 91 | 85 | 79 | 77 | 72 |
|  | 0.336 | 135 | 122 | 114 | 104 | 97 | 90 | 86 | 78 | 71 | 67 | 62 | 59 |
|  | 0.448 | 143 | 127 | 117 | 107 | 96 | 89 | 82 | 75 | 67 | 62 | 56 | 51 |
|  | 0.560 | 158 | 137 | 125 | 114 | 103 | 92 | 88 | 78 | 69 | 61 | 57 | 51 |
|  | 0.672 | 189 | 161 | 143 | 128 | 112 | 100 | 92 | 80 | 70 | 62 | 56 | 49 |
| Fe mmol/L | |  | | | | | | | | | | | |
| Ferritin | 3.76 | 82 | 78 | 76 | 76 | 72 | 70 | 71 | 68 | 65 | 63 | 61 | 60 |
|  | 5.37 | 60 | 56 | 55 | 53 | 52 | 51 | 48 | 48 | 45 | 44 | 44 | 43 |
|  | 6.98 | 59 | 57 | 53 | 54 | 51 | 49 | 49 | 47 | 45 | 43 | 42 | 41 |
|  | 8.60 | 78 | 74 | 70 | 68 | 64 | 63 | 64 | 61 | 56 | 54 | 52 | 52 |
|  | 10.21 | 114 | 109 | 102 | 100 | 94 | 93 | 92 | 88 | 82 | 81 | 77 | 76 |
| mol/L | |  | | | | | | | | | | | |
| CaCl_2_ | 0.9 | 67 | 65 | 63 | 64 | 60 | 59 | 60 | 60 | 56 | 55 | 54 | 54 |
|  | 1.8 | 46 | 47 | 43 | 45 | 42 | 42 | 41 | 41 | 38 | 38 | 36 | 37 |
|  | 2.7 | 49 | 47 | 46 | 47 | 44 | 44 | 44 | 42 | 40 | 39 | 38 | 38 |
|  | 3.6 | 66 | 63 | 60 | 59 | 56 | 56 | 54 | 53 | 49 | 50 | 48 | 46 |
|  | 4.5 | 91 | 86 | 82 | 82 | 75 | 75 | 72 | 70 | 65 | 63 | 59 | 60 |
| mol/L | |  | | | | | | | | | | | |
| CaCO_3_ | 1.0 | 92 | 89 | 82 | 76 | 72 | 65 | 59 | 54 | 50 | 47 | 43 | 37 |
|  | 2.0 | 67 | 59 | 48 | 41 | 34 | 26 | 23 | 19 | 15 | 13 | 12 | 10 |
|  | 3.0 | 53 | 46 | 38 | 29 | 25 | 21 | 16 | 14 | 9 | 12 | 6 | 7 |
|  | 4.0 | 59 | 46 | 37 | 25 | 20 | 11 | 10 | 9 | 8 | 7 | 5 | 5 |
|  | 5.0 | 70 | 54 | 36 | 24 | 18 | 13 | 10 | 8 | 7 | 5 | 6 | 6 |

| Table S1 (b) | | TE1: 3.15 | TE2: 6.3 | TE3: 9.45 | TE4: 12.6 | TE5: 15.75 | TE6: 18.9 | TE7: 22.05 | TE8: 25.2 | TE9: 28.35 |
| --- | --- | --- | --- | --- | --- | --- | --- | --- | --- | --- |
| Fe mmol/L | |  | | | | | | | | |
| USPIO | 0.224 | 154 | 142 | 128 | 116 | 106 | 96 | 87 | 79 | 72 |
|  | 0.336 | 160 | 141 | 124 | 108 | 96 | 83 | 74 | 65 | 57 |
|  | 0.448 | 173 | 148 | 125 | 105 | 90 | 76 | 66 | 57 | 47 |
|  | 0.560 | 159 | 134 | 113 | 93 | 80 | 68 | 57 | 47 | 40 |
|  | 0.672 | 139 | 113 | 91 | 73 | 61 | 50 | 40 | 32 | 28 |
| Fe mmol/L | |  | | | | | | | | |
| Ferritin | 3.76 | 87 | 82 | 78 | 73 | 69 | 64 | 61 | 58 | 56 |
|  | 5.37 | 90 | 84 | 79 | 73 | 70 | 65 | 62 | 58 | 55 |
|  | 6.98 | 98 | 90 | 82 | 74 | 70 | 64 | 58 | 54 | 50 |
|  | 8.60 | 102 | 92 | 82 | 74 | 68 | 60 | 56 | 50 | 45 |
|  | 10.21 | 96 | 85 | 76 | 68 | 62 | 54 | 50 | 44 | 40 |
| mol/L | |  | | | | | | | | |
| CaCl_2_ | 0.9 | 101 | 98 | 95 | 92 | 90 | 87 | 85 | 81 | 79 |
|  | 1.8 | 84 | 82 | 78 | 74 | 74 | 72 | 69 | 66 | 64 |
|  | 2.7 | 90 | 85 | 79 | 76 | 71 | 68 | 64 | 61 | 57 |
|  | 3.6 | 99 | 91 | 87 | 81 | 78 | 74 | 70 | 66 | 63 |
|  | 4.5 | 128 | 115 | 100 | 93 | 81 | 77 | 65 | 64 | 53 |
| mol/L | |  | | | | | | | | |
| CaCO_3_ | 1.0 | 58 | 38 | 26 | 18 | 14 | 12 | 10 | 10 | 9 |
|  | 2.0 | 43 | 21 | 12 | 9 | 9 | 9 | 9 | 9 | 9 |
|  | 3.0 | 38 | 17 | 10 | 9 | 9 | 9 | 9 | 9 | 9 |
|  | 4.0 | 28 | 11 | 9 | 9 | 9 | 9 | 9 | 9 | 9 |
|  | 5.0 | 21 | 9 | 8 | 9 | 8 | 8 | 8 | 9 | 8 |

Table S1: SNR at each TE for each material concentration, at (a) 3T, and (b) 7T, respectively. The SNR ranges were defined as follows; high SNR (in green): SNR ≥ 50:1, intermediate SNR (in yellow): 20 ≤ SNR < 50, borderline (in orange): 10 ≤ SNR < 20, low (in red): <10.

|  | USPIO | Ferritin | CaCl_2_ | CaCO_3_ |
| --- | --- | --- | --- | --- |
| Volume of elevated R_2_^*^ (mm^3^) | 3.27 ± 3.08; 6.40 ± 2.26 | 3.49 ± 3.22; 13.01 ± 15.73 | 1.46 ± 0.82; 5.59 ± 2.72 | 1.27 ± 0.60; 10.40 ± 8.83 |
| Voxels of elevated R_2_^*^ (% per vial) | 2.65 ± 0.65;  0.14 ± 0.15 | 1.26 ± 0.35; 1.92 ± 0.77 | 1.00 ± 0.07; 0.47 ± 0.01 | 0.08 ± 0.09; 0.14 ± 0.14 |

Table S2: Volume of elevated R_2_^*^ and voxels of elevated R_2_^*^ at 3 T; 7 T. All values were reported as mean ± standard deviation.

|  | USPIO | Ferritin | CaCl_2_ | CaCO_3_ |
| --- | --- | --- | --- | --- |
| R_2_^*^_mol_ (s^-1^·L·mmol^−1^) | 84.2 ± 7.9;  79.5 ± 4.4 | 0.77 ± 0.16;  2.78 ± 0.41 | 10^-4^× (-1.83 ± 3.38);  10^-4^× (8.60 ± 2.52) | 10^-2^× (3.37 ± 0.35);  10^-2^× (8.69 ± 4.99) |
| R_2_^*^_0_ (s^-1^) | 17.0 ± 2.6 (p = 0.14); 16.2 ± 1.1 (p = 0.00) | 13.3 ± 1.1 (p = 0.63);  8.5 ± 2.6 (p = 0.73) | 14.4 ± 0.8 (p = 0.00);  12.7 ± 0.6 (p = 0.00) | 18.6 ± 11.4 (p = 0.20);  38.7 ± 34.0 (p = 0.37) |
| R^2^ (c_mol_ versus R_2_^*^) | 0.97; 0.99 | 0.92; 0.93 | 0.13; 0.85 | 0.97; 0.95 |

Table S3: Linear fitting coefficients (c_mol_ versus R_2_^*^) and linearity (R^2^) at 3T; 7T, respectively. Coefficients were reported with standard error.

|  | USPIO | Ferritin | CaCl_2_ | CaCO_3_ |
| --- | --- | --- | --- | --- |
| χ_3T_ versus χ_7T_ ** | 0.61 ± 0.16 A/m (p = 0.00) | -0.02 ± 0.01 ppm (p = 0.00) | -0.01 ± 0.02 ppm (p = 0.12) | -0.01 ± 0.01 ppm (p = 0.02) |
| (R_2_^*^)_3T_ versus (R_2_^*^)_7T_ ** | -2.2 ± 2.3 s^-1^ (p = 0.01) | -2.1 ± 0.7 s^-1^∙T^-1^ (p = 0.00) | 0.8 ± 1.5 s^-1^ (p = 0.12) | -3.8 ± 11.3 s^-1^∙T^-1^ (p = 0.22) |

Table S4: Bland-Altman analysis (bias ± limit-of-agreement). The results of a t-test were shown using zero bias as the null hypothesis. ** M_3T_ versus M_7T_ was assessed for USPIO, and (R_2_^*^/B_0_)_3T_ versus (R_2_^*^/B_0_)_7T_ was assessed for CaCO_3_ and ferritin.

|  | CaCO_3_ (mol/L) | 1.0 | 2.0 | 3.0 | 4.0 | 5.0 | R^2^ |
| --- | --- | --- | --- | --- | --- | --- | --- |
| 3T | χ_all-TE_ | 81 ± 40 | -34 ± 19 | -56 ± 19 | -180 ± 23 | -346 ± 31 | 0.94 |
|  | χ_excl-TE_ | 83 ± 40 | -32 ± 17 | -55 ± 19 | -174 ± 21 | -333 ± 35 | 0.94 |
| 7T | χ_all-TE_ | 72 ± 70 | -39 ± 40 | -63 ± 26 | -63 ± 16 | -19 ± 16 | 0.19 |
|  | χ_excl-TE_ | 75 ± 64 | -53 ± 47 | -84 ± 28 | -190 ± 46 | -335 ± 68 | 0.96 |

Table S5: Susceptibility value (in ppb units) at CaCO_3_ ROIs derived from susceptibility maps reconstructed with all echo times (χ_all-TE_) and with later echo times excluded (χ_excl-TE_). Coefficient of determination (R^2^) was used to evaluate the goodness-of-fit between molar concentration and susceptibility.

# References

1. Barick KC, Aslam M, Lin YP, Bahadur D, Prasad PV, Dravid VP. Novel and efficient MR active aqueous colloidal Fe3O4 nanoassemblies. *Journal of Materials Chemistry*. 2009;19(38):7023-7029.

2. Bullivant JP, Zhao S, Willenberg BJ, Kozissnik B, Batich CD, Dobson J. Materials Characterization of Feraheme/Ferumoxytol and Preliminary Evaluation of Its Potential for Magnetic Fluid Hyperthermia. *International Journal of Molecular Sciences*. 2013;14(9):17501-17510.

3. Schenck JF. Health and Physiological Effects of Human Exposure to Whole-Body Four-Tesla Magnetic Fields during MRI. *Annals of the New York Academy of Sciences*. 1992;649(1):285-301.

4. Vuong QL, Gillis P, Roch A, Gossuin Y. Magnetic resonance relaxation induced by superparamagnetic particles used as contrast agents in magnetic resonance imaging: a theoretical review. *WIREs Nanomed Nanobiotechnol*. 2017;9(6):e1468. doi:10.1002/wnan.1468
